# Evidence that human and equine erythrocytes could have significant roles in the transport and delivery of amino acids to organs and tissues

**DOI:** 10.1007/s00726-020-02845-0

**Published:** 2020-04-21

**Authors:** B. Thorn, R. H. Dunstan, M. M. Macdonald, N. Borges, T. K. Roberts

**Affiliations:** School of Environmental and Life Sciences, University of Newcastle, Callaghan, Australia

**Keywords:** Erythrocytes, RBC, Red blood cells, Amino acids, Transport, Inter-organ transport

## Abstract

Erythrocytes have a well-defined role in the gaseous exchange of oxygen and carbon dioxide in the mammalian body. The erythrocytes can contain more than half of the free amino acids present in whole blood. Based on measures showing that venous erythrocyte levels of amino acids are much less than arterial erythrocyte levels, it has previously been proposed that erythrocytes also play a role in the delivery of amino acids to tissues in the body. This role has been dismissed because it has been assumed that to act as an amino acid transport vehicle, the erythrocytes should release their entire amino acid content in the capillary beds at the target tissues with kinetic studies showing that this would take too long to achieve. This investigation set out to investigate whether the equine erythrocytes could rapidly take up and release smaller packages of amino acids when exposed to high or low external concentrations of amino acids, because it seemed very unlikely that cells would be able to release all of their amino acids without serious impacts on osmotic balance. Freshly prepared erythrocytes were placed in alternating solutions of high and low amino acid concentrations in PBS to assess the capacities of these cells to rapidly take up and release amino acids depending on the nature of the external environment. It was found that amino acids were rapidly taken up and released in small quantities in each cycle representing 15% of their total load in equine erythrocytes and 16% in human erythrocytes. The capacity for rapid uptake/release of amino acids by equine and human erythrocytes provided evidence to support the theory that mammalian erythrocytes have a significant role in transport of amino acids from the liver to tissues, muscles and organs.

**Electronic supplementary material:**

The online version of this article (10.1007/s00726-020-02845-0) contains supplementary material, which is available to authorized users.

## Introduction

Fully matured erythrocytes do not contain the organelles that were required for development during erythropoiesis in precursor reticulocytes. Consequently, the erythrocyte does not undergo oxidative respiration, or is it capable of de novo synthesis of proteins for repair and maintenance. However, the cells do undergo glycolysis producing lactic acid and they can synthesise glutathione for antioxidant protection (Ataullakhanov et al. 1981; Wu et al. 2004). Due to the inability to repair or produce proteins, erythrocytes have a limited life span in circulation and, once damaged or aged, are removed from the vascular system and terminated in a process known as eryptosis (Klei et al. 2017; Lang et al. 2012). In horses, the life span of the erythrocytes is 140–150 days (Carter et al. 1974) and in humans, this is averaged at around 115 days (Franco 2012). The cytoplasm of the mature erythrocyte is rich in haemoglobin (Hb), a metalloprotein capable of efficiently binding oxygen (Dominguez de Villota et al. 1981; Weed et al. 1963). Consequently, the primary roles ascribed to the erythrocyte in circulation are to carry oxygen to the tissues, muscles, and organs and also to transport CO_2_ to the lungs for exhalation (Mangum 1992; Scholander 1960).

Despite these primary roles, evidence from as early as 1913 and 1917 had showed that animals, birds and humans had higher levels of amino acids in erythrocytes compared with their corresponding serum levels (Bock 1917; Costantino 1913) which was confirmed more recently in humans (Agli et al. 1998). Costantino also provided the first evidence that the erythrocytes were permeable to amino acids (Costantino 1913), whereas Winter and Christensen performed some of the first kinetic studies investigating amino uptake systems in the erythrocyte (Winter and Christensen 1964). The limited metabolism in erythrocytes and the absence of protein synthesis mandates that the erythrocytes themselves have no intrinsic requirement for a full array of amino acids in the cytoplasm. If amino acids are present in high concentrations in the erythrocytes without being required by internal metabolic processes, then a logical explanation would involve the erythrocyte acting as an inter-organ amino acid transport vessel. The results from Winter and Christensen (1964) effectively showed that the erythrocyte levels of amino acids were not completely depleted in a short time frame and some amino acids continued to be released from the erythrocyte over 120 min. On the basis of “The slow equilibration time of amino-acid transport across erythrocyte membranes”, it was “generally believed that plasma rather than erythrocytes is the vehicle of amino-acid exchange between tissues” (Felig et al. 1973). Consequently, pathologic investigations have generally focussed on measuring plasma levels of amino acids, because plasma has been portrayed as the vehicle of inter-organ transport in textbooks (Munro 1970), reviews (Brosnan 2003) and encyclopaedias (Schwartz and Conley 2019).

Despite the consensus at the time, research into the potential role of erythrocytes as inter-organ transporters continued. Amino acid concentrations were measured in canine plasma and erythrocytes during transit from the gastro-intestinal tract (GIT) through the portal vein to the liver (Elwyn et al. 1968, 1972). It was found that the amino acids were in greater abundance in the plasma than the erythrocytes. In the hepatic vein bringing blood from the liver to the vena cava of the heart and ultimately to other organs, the opposite was observed—amino acids were concentrated primarily in the erythrocytes. In addition, the amino acid exchange rates for plasma to liver and liver to erythrocytes were much higher than exchange rates from erythrocytes to plasma and the transfer rates were a direct function of blood flow and amino acid concentrations. It was concluded that amino acids were being exchanged between plasma and liver tissue cells as well as between liver tissue cells and erythrocytes and that these exchanges were independent of each other.

In 1973, Felig and colleagues published a landmark paper that showed that quantities of amino acids transported by plasma alone could not account for the total amino acid concentrations absorbed from digestion of proteins in the gastrointestinal tract and splanchnic bed in humans (Felig et al. 1973). These authors demonstrated that transport of amino acids by erythrocytes was responsible for 22–32% of the net movement of alanine into and out of the whole blood in a range of tissues. In addition, it was shown that amino acid levels in erythrocytes were lower in venous *vs* arterial samples indicating that amino acids had been removed from the erythrocytes during passage through the capillary beds. Other studies from the similar era concluded that “circulating blood cells have a dynamic role in glutamate transport” (Aoki et al. 1975) with a “more rapid transfer rate on passage of blood through the capillary bed” (Felig et al. 1973). Together, these observations provided strong evidence that erythrocytes act as an inter-organ transport vehicle for amino acids (Elwyn et al. 1968, 1972; Felig et al. 1973). In this model, the liver would primarily receive post-absorptive amino acids from the GIT as well as those released from endogenous protein turnover in the muscles via the plasma. Subsequently, the liver would transfer the amino acids to the erythrocytes on demand for delivery of amino acids to target muscles, organs and tissues.

The ability to transport amino acids across the membrane of erythrocytes has been shown to be facilitated by at least seven transporter proteins (Tunnicliff 1994). Three of the amino acid transporters have been characterised as secondary active transport systems (Ellory et al. 1981; Ellory and Osotimehin 1984; Young et al. 1979) and the remaining four have been identified as operating via facilitated diffusion (Deves et al. 1992; Gardner and Levy 1972; López-Burillo et al. 1985; White 1985). The presence of facilitated diffusion and secondary active transport systems would generate a capacity for the erythrocyte to accumulate amino acids at concentrations different to those observed in the plasma. It was shown that erythrocytes and plasma have very different profile characteristics where at least eight amino acids were measured at significantly higher levels in the erythrocytes than the corresponding plasma and two amino acids (glutamine and arginine) were found to be lower (*p* < 0.01) (Agli et al. 1998). Aspartic acid showed contrasting levels in erythrocytes and plasma analysed from resting blood samples, with 451–484 µM in the erythrocytes compared with 14.5–15.1 µM in the corresponding plasma (MacLaren et al. 2000).

Amino acid levels in human plasma and erythrocytes were found to increase significantly in the post-absorptive state following an oral loading of amino acids (Agli et al. 1998). A separate study in a human exercise challenge demonstrated that erythrocytes increased their carrying capacity of amino acids during exercise with no corresponding change in the plasma levels (MacLaren et al. 2000). These investigations have made important and relevant contributions providing evidence that the amino acid concentrations in erythrocytes are acutely sensitive to lifestyle interventions and alter post-absorption or in response to exercise.

The equilibration times of amino acids into and out of erythrocytes were deemed “slow” on the basis of considering release or uptake of the whole erythrocyte load capacity. This conclusion proved to be a key limiting factor preventing progress and development of this theory; no later work considering erythrocytes as carrier vessels for amino acids could be found. New insight into understanding the potential role of erythrocytes in amino acid transport could arise by considering that erythrocytes may be limited to rapidly releasing only small quantities of amino acids at any one time. The full, rapid release of the erythrocyte load of amino acids at one time would be unlikely, because it would cause great disruption to cellular osmotic balance. If the erythrocytes were able to release—and take up—smaller quantities of amino acids over a very short time frame comprising seconds, then this would be consistent with the earlier investigations of erythrocyte transport kinetics (Winter and Christensen 1964). Therefore, the current study investigated equine erythrocytes over short time frames to determine whether these cells could release amino acids into a medium without amino acids and conversely take them up from a medium with high levels of amino acids. The aim was to investigate whether a population of erythrocytes could alternate cycles of uptake and release in vitro in successive exposures to a “liver environment” (relatively higher amino acids concentrations) and a “muscle/tissue environment” (relatively lower amino acids concentrations). The same experimental approach was applied to human erythrocytes to determine whether this capacity could be confirmed in another mammalian species.

## Methods

The blood used for all experiments on equine erythrocytes came from a single horse and was taken at defined stages throughout a training regime of preparation for harness training. The horse had been rested for 6 months prior to initiating fitness training as part of a larger study. The fitness program is summarised in Table 1; there was an initial jogging phase for four weeks followed by a progressive phase of fast-work training for a further 8 weeks. By the end of the fast-work phase, the horse was deemed race-fit. The pre-exercise blood samples were collected for testing after 2 and 4 weeks of jogging training and then after 2, 9, 13, 16 and 19 weeks of fast-work training. In this way, any potential changes in erythrocyte and plasma amino acid levels over the training program could be assessed.

**Table 1:** Summary of the fitness-training schedule for the Standardbred horse

Preparation	Weeks	Schedule
Jogging phase	0–2 3–4	Jog for 3 km per day working up to 9 km per day by the end of the two week period Maintain 9 km per day for a further two weeks
Fast work phase	0–4	Start slow pace work or cantering every second day, gradually building to half and ¾ maximum intensity; no full-speed work
	5–6	Speed work integrated every 3 days over 2.8 km for 3 min and 50 s to 4 min and 10 s, whilst wearing a trotting harness; 9 km jog on days in-between. One day off in seven
	7–8	Horse deemed ready for pre-racing trials by the end of week 8
Racing	9–19	Periodic hard-work sessions maintained 2–3 times per week integrated with racing

Fresh whole blood samples were taken from the Standardbred horse prior to training and feeding in the early morning. Venous blood from the jugular vein was collected in Vacuette^®^ lithium heparin tubes (9 mL) and chilled for transport to the laboratory. Approval was received from the University of Newcastle Animal Care and Ethics Committee (approval number A-2017-707).

A single resting blood sample was collected from one healthy human male participant, who averaged 5 h exercise per week and was recruited from the Newcastle University campus (aged 59 years, 1.78 m, 92 kg, no medications). The human blood sample was collected from the antecubital vein participant by venepuncture in Vacuette^®^ lithium heparin tubes (9 mL) following an overnight fast. Approval was received from the University of Newcastle Human Research Ethics Committee (approval number H-2018-0314) and the participant provided written informed consent. Whole blood samples were centrifuged at 2000×*g* for 15 min to separate the blood cells from the plasma. The plasma and ‘buffy’ layer of white blood cells were removed, and the remaining isolated erythrocytes were washed three times in sterile phosphate buffered saline (PBS, pH 7.3) to remove residual plasma/white blood cell constituents. All experiments were conducted on the same day as sample collection.

A medium comprising phosphate-buffered saline (PBS) with amino acids at concentrations higher than the average levels measured in horse erythrocytes (except alanine) was designed as a loading medium (“liver environment”) to provide amino acids to erythrocytes (AA-PBS). Alanine was provided at a level less than the average erythrocyte concentration to see if it could be taken up by the erythrocyte. PBS (without amino acids) was used to incubate erythrocytes to determine whether amino acids could be released from erythrocytes (“muscle/tissue environment”). To determine the average concentrations of amino acids in the erythrocytes, sample extracts were used to calculate the average concentrations in the erythrocytes. The final concentrations of amino acids included in the medium are summarised in Table 2 for comparison against the average values for the horse used as a source of erythrocytes and an additional two horses at similar levels of fitness. The osmolality of the AA-PBS solution was calculated to ensure that it was within a tolerable range for the erythrocytes. Cystathionine (CTH) was not present in high abundance in the erythrocytes but was initially included in high concentration in the AA-PBS to determine whether there was a general gradient-driven uptake of amino acid substrates. However, due to inconsistent results with the measurements of cystathionine throughout the study, further evaluations of this amino acid were conducted with GC–MS. It was revealed that the peak for cystathionine was not homogeneous and thus this amino acid was not included in the final analyses in this study. The calculated total of amino acids in AA-PBS was 6522 μM. The actual level of amino acids was measured by GC-FID to account for compounding errors in the preparation of the complex medium and losses incurred during autoclaving, resulting in a measured total level of 5548 μm.

**Table 2:** Average concentrations of key amino acids found in equine erythrocytes (averaged from three horses) and corresponding higher concentrations required for the AA-PBS loading medium for the “liver environment”

Amino acid	Average concentration in horse (µM)	Concentration added to AA-PBS (µM)
GLY	587	869
SER	448	862
VAL	439	854
THR	331	655
PRO	318	652
LYS	303	608
ALA	274	147
ORN	165	234
ASP	123	216
LEU	108	252
HIS	107	220
GLN	79	172
ILE	59	123
PHE	43	85
MET	24	58
TRP	23	33
CTH	0	482

The initial erythrocyte amino acid concentrations were determined by transferring 100 µL of the washed erythrocytes to a clean Eppendorf tube containing 100 µL of Milli-Q H_2_O (ratio 1:1) to induce lysis. This was stored on ice and the remainder of the washed erythrocyte preparation used to generate samples for incubation either with or without a loading of amino acids. Incubating erythrocytes were resuspended in either pre-warmed PBS or fresh AA-PBS to generate a haematocrit of 0.44 L/L and sampled in triplicate at collection periods of 2, 5, 10 and 20 min.

To assess whether erythrocytes were capable of repetitive cycles of uptake and release of amino acids, equine erythrocytes from the horse in the fast work stage of preparation were consecutively exposed to media with high (AA-PBS) and zero (PBS) levels of amino acids. Fresh erythrocytes were washed before suspension in pre-warmed (37 °C) PBS for 2 min. Cells were then centrifuged at 1500×*g* for 1 min, the supernatant removed for analysis and the cells transferred to pre-warmed AA-PBS medium for a 2-min incubation. This process was repeated, alternating cycles of exposure to PBS and AA-PBS media for 2-min incubation periods in a sequence comprising nine cycles (five PBS incubations and four AA-PBS incubations). Aliquot samples of erythrocytes and external media were taken at the end of each incubation cycle. Erythrocyte amino acid levels were measured initially and at the end of each cycling step. Each complete cycling series was performed in triplicate on the same source of erythrocytes within 3 h (*n* = 3). Human erythrocytes were exposed to the same series of cycling incubations.

Lysis of washed erythrocytes was achieved by removing 100 µL of the cell pellet and mixing with an equal volume of Milli-Q H_2_O (ratio 1:1). The erythrocyte lysate contained a high level of protein which caused issues in the ion exchange preparations for extraction of amino acids. A range of procedures ranging from HCl, acetonitrile/methanol/H_2_O 2:2:1 and 3% 5-sulfosalicylic acid precipitations were tested as described in the supplementary information provided in Online Resources 1. The optimal extraction procedure ultimately involved no protein precipitation but included a filtration step. The lysate was vortexed, rested for 5 min, vortexed once more, then centrifuged at 15,000×*g* for 5 min. The cytoplasmic lysate was filtered by transferring to QIAgen spin columns and centrifuging at 15,000×*g* for 5 min. The filtered lysate (100 µL) was added to 200 µL Milli-Q H_2_O with 100 μL norvaline as the internal standard and processed using EZ:Faast™ (Phenomenex^®^ Inc.) derivatisation kits for amino acid analysis by gas chromatography with flame ionization detection (GC/FID) as previously described (Dunstan et al. 2015). The EZ:Faast™ kit has been designed for rapid and efficient analyses of amino acids in plasma (Badawy 2019; Badawy et al. 2008). The GC-FID was calibrated with reference standards of each amino acid at levels of 5, 10, 20, 30 and 40 nmoles/100 μL with correlations of  > 0.99 for all amino acids except valine which had *r* = 0.984 (see Online Resource 2). The capacity to measure appropriate quantities of amino acids in the erythrocyte extracts was assessed by comparisons of levels measured in the literature from prior equine and human studies shown in Online Resource 3. It was noted that the amino acids in the erythrocytes were highly variable between studies and groups within a single study, most likely reflecting genetics, nutrition, age, fitness and other variables. This was dealt with in the current study by always measuring the Time 0 level of amino acids in the erythrocytes as part of the experimental design.

Statistica (TIBCO Software Inc. 2017, version 13) was used for all analysis of variance (ANOVA), Tukey HSD, and Duncan’s multiple range tests to determine statistical significance when comparing data. Results were considered statistically significant at *p* < 0.05.

## Results

Blood was collected and analysed for amino acid composition from one horse throughout a program of fitness training to become race fit and was used to evaluate the capacity of erythrocytes to take up and release amino acids. The amino acid status of the erythrocytes was monitored throughout the period of experimentation. Resting pre-exercise blood samples were collected twice during the initial jogging training and five times during the period of fast-work training and racing. Corresponding plasma was measured after 4 weeks jogging and again at 2 weeks, 9 weeks and 13 weeks fast work.

**Fig. 1:**
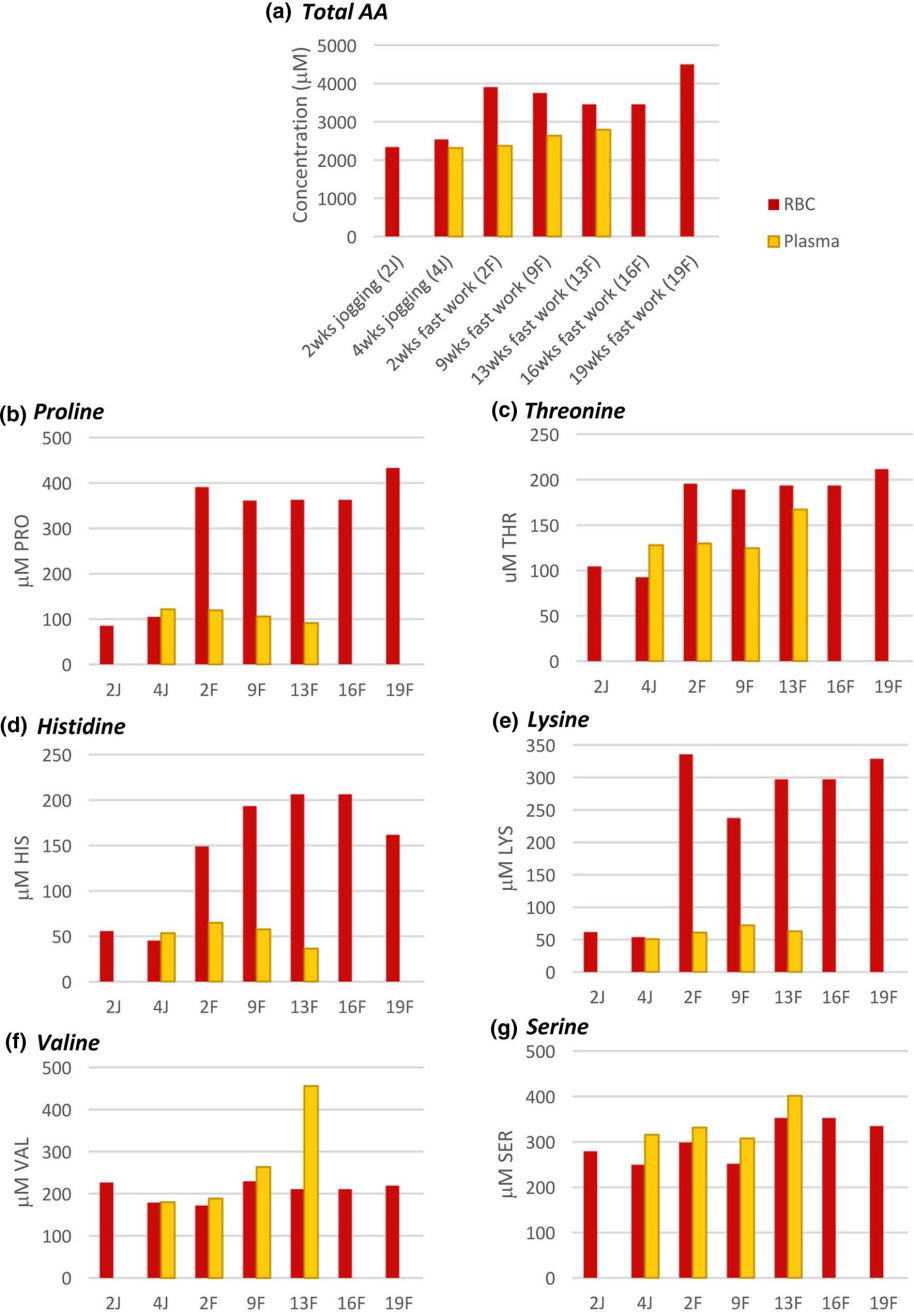
**a** Concentrations of total amino acids were measured in the erythrocytes and plasma from one horse during a training regime of 23 weeks (from unfit to race-fit). Examples of individual analysis of **b** proline, **c** threonine, **d** histidine, **e** lysine, **f** valine, and **g** serine have been presented

The measurements of the total concentrations of amino acids in the erythrocytes indicated that the levels increased from 2500 µM during the 4 weeks of jogging to over 3400 µM during the period of fast work reaching 4500 µM after 19 weeks (Fig. 1a). The total plasma amino acid concentration did not increase in the first 2 weeks of fast work but did show a small but progressive increase of 400 µM over the period from 2–13 weeks of fast work. Evaluation of individual amino acid concentrations showed specific alterations in erythrocyte amino acids over the training period (Fig. 1b–g). For example, levels of proline (b), threonine (c), histidine (d) and lysine (e) all increased in erythrocytes during the fast work training whilst the plasma concentrations remained relatively constant, although an increase in plasma threonine was noted at week 13. Valine (f) and serine (g) maintained relatively constant in both the erythrocytes and the plasma throughout the jogging and first 9 weeks of fast-work training but both amino acids showed increased levels in plasma at week 13.

**Fig. 2:**
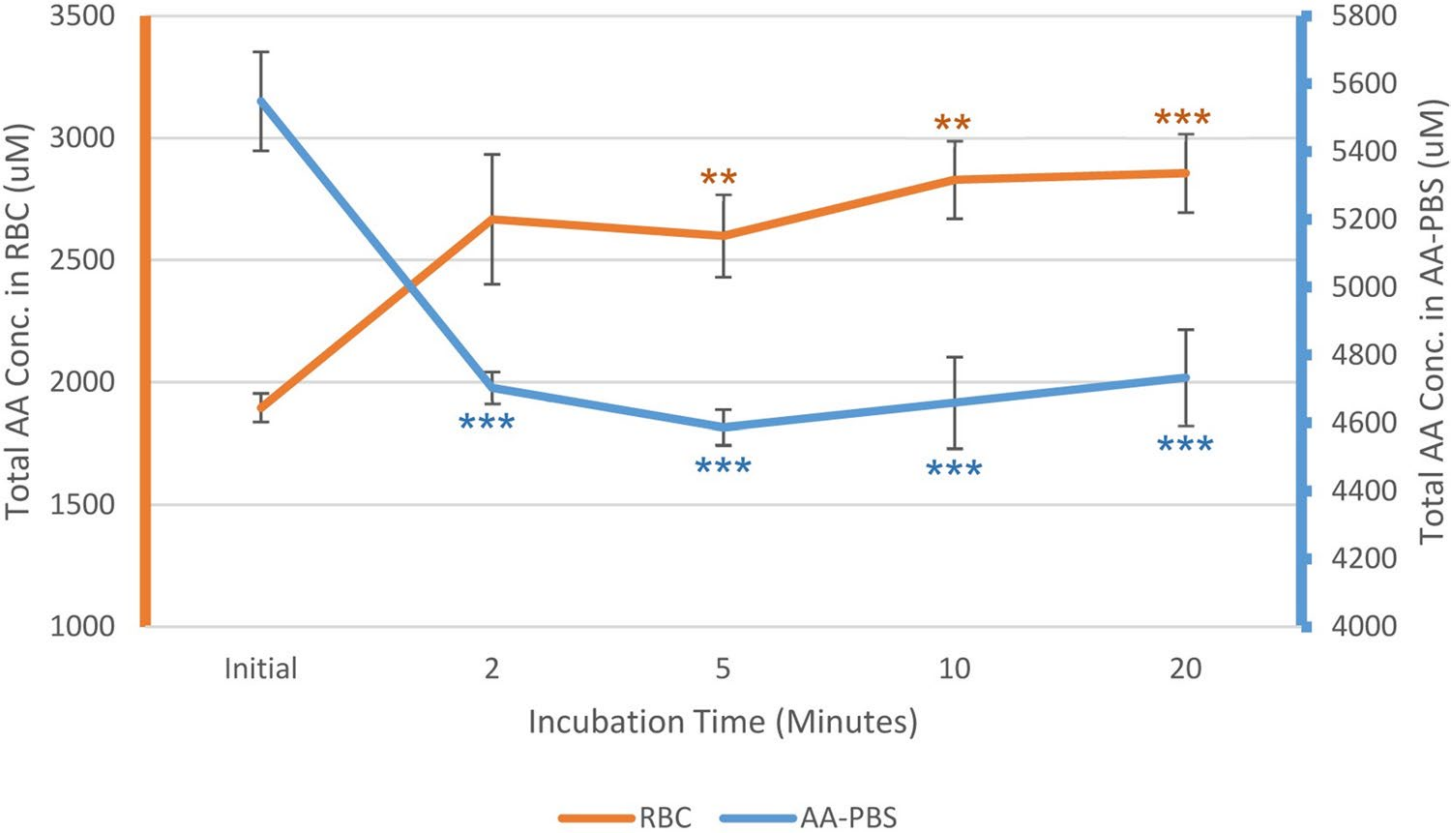
The total levels of amino acids in freshly prepared equine erythrocytes at time 0 and then after 2, 5, 10, and 20 min incubation in pre-warmed AA-PBS (orange, mean ± SE, *n* = 3). The corresponding levels of amino acids in the external AA-PBS medium were also assessed (blue, *n* = 3). Significant differences were marked between the initial value and the subsequent time points; **p < 0.001; ***p < 0.0001

Using blood taken from the horse in the jogging phase of training, the ability for equine erythrocytes to take up amino acids from their external environment into their cytoplasm was investigated by comparing total amino acids in freshly prepared erythrocytes with levels after incubation for 2, 5, 10 and 20 min in pre-warmed (37 °C) PBS medium containing amino acids (AA-PBS). The total starting concentration of amino acids in the AA-PBS was 5548 µM and the initial average total concentration of amino acids in the erythrocytes was 1895 µM (Fig. 2).

After 2-min incubation, the concentration of total amino acids in the AA-PBS medium dropped by 845 µM to 4,703 ± 115 µM (*p* < 0.0001) and the concentration of amino acids in the erythrocytes increased by 772 µM to 2,667 ± 217 µM. The total amino acid levels remained relatively stable between 2599 and 2856 µM over the remaining 20 min. The values at 5, 10 and 20 min were significantly higher than the initial value (*p* < 0.01) but there were no further significant alterations between the 2-, 5-, 10-, and 20-min samples. In parallel, the loss of amino acids from the AA-PBS occurred within the first 2 min of incubation (*p* < 0.001) with no further significant alterations between the 2, 5, 10, and 20-min samples.

**Fig. 3:**
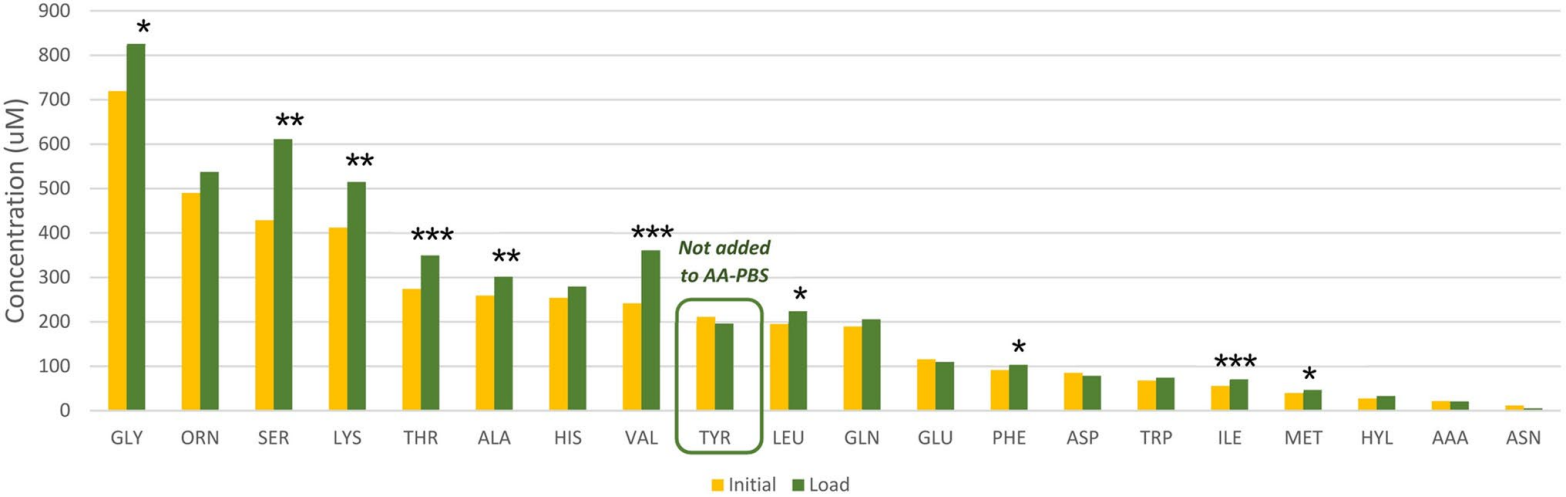
The concentrations of individual amino acids measured in equine erythrocytes before (yellow) and after (green) incubation at 37 °C for 2 min in the medium loaded with high concentrations of amino acids in the AA-PBS medium (*n* = 3). Significant differences are marked for comparisons between the initial and 2-min post-loading values, *p < 0.05, **p < 0.01, ***p < 0.001

To ascertain if all or only a selection of the amino acids provided were taken up by the erythrocytes from the AA-PBS medium, the concentrations of the twenty most abundant amino acids in the equine erythrocytes were assessed after the 2-min incubation period. The amino acid profiles for the initial pre-incubation erythrocyte amino acids, Time 0, were compared with those recorded after the 2 min incubation in AA-PBS medium (Fig. 3). It was observed that valine increased significantly by 50%, serine 43%, isoleucine 29%, lysine 25%, threonine 25%, methionine 19%, alanine 16%, glycine 16%, leucine 15% and phenylalanine 14% (*p* < 0.05). Notably, some of the amino acids in the AA-PBS medium, including ornithine, histidine, glutamine and tryptophan, were not taken up by the erythrocytes under these conditions. Alanine was present in the AA-PBS medium at a lower concentration than the starting cell content, but had a higher concentration in the erythrocyte after incubation. Tyrosine was not present in the AA-PBS and correspondingly showed no increase in the erythrocytes after incubation. Even though the peak for cystathionine was shown to be heterogeneous (see “Methods”), there was no evidence of this peak in extracts from cells after incubation in AA-PBS and, therefore, no evidence of cystathionine entering the cells from the AA-PBS.

**Fig. 4:**
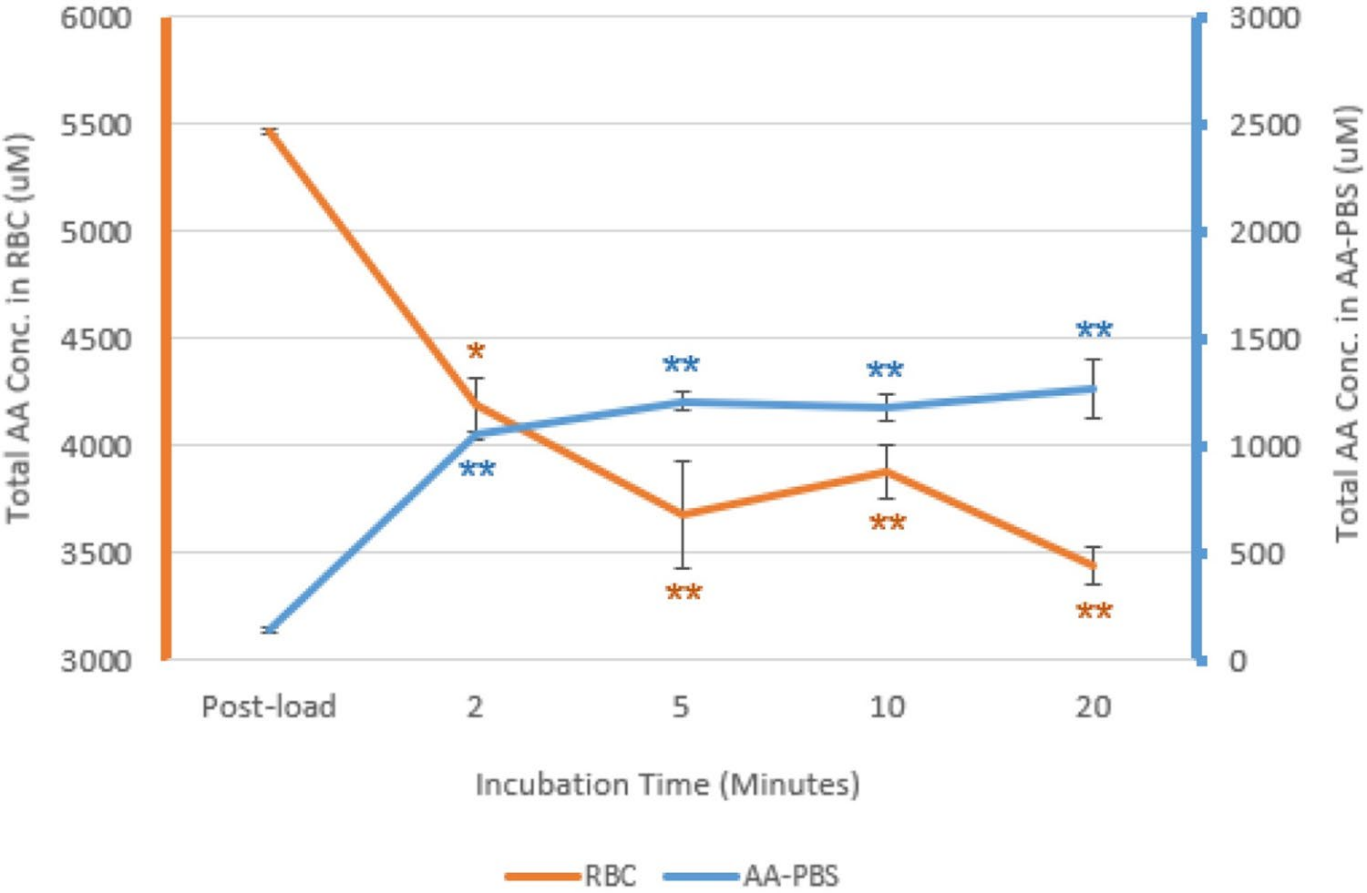
Concentration of total equine erythrocyte cytoplasmic amino acids (orange) and corresponding external media (blue) for erythrocytes which had been pre-loaded with amino acids prior to washing and incubation in PBS at 37 °C (mean ± SE, *n* = 3). Significant differences were marked between the initial value and the subsequent time points *p < 0.01; **p < 0.001

Having demonstrated that equine erythrocytes can take up amino acids from their external medium within 2 min, the capacity of these “loaded” cells to subsequently release amino acids back into an external medium that had no amino acids was investigated. The remaining blood samples were taken from the horse during the fast-work phase of training where the resting erythrocyte levels of amino acids were higher than the samples from the jogging phase. Fresh erythrocytes were pre-loaded with amino acids by an initial incubation in AA-PBS at 37 °C for 2 min. Even though a higher starting level of amino acids were present in the erythrocytes, a similar magnitude of increase of 771 µM total amino acids was observed in erythrocytes following exposure to the AA-PBS, reaching a level of 5,463 µM (*p* < 0.0001). These preloaded cells were centrifuged, washed and transferred to fresh PBS for a further incubation at 37 °C with samples taken at time 0, 2, 5, 10, and 20 min. After 2-min incubation at 37 °C in PBS, total amino acids levels in the erythrocytes decreased (*p* < 0.05) and conversely, the concentrations of amino acids in the PBS medium increased (*p* < 0.05) as shown in Fig. 4. In the next 18 min of incubation, the erythrocyte amino acids concentrations continued to drop less rapidly and amino acids in the PBS medium showed smaller increases.

To test the hypothesis that equine erythrocytes can act as transport vehicles delivering amino acids to target tissues, the capacity to take-up and release amino acids repeatedly in response to changing external environments was investigated using a population of washed cells exposed to alternating incubations in AA-PBS medium and PBS (amino acid-free medium). Cell and media samples were analysed at each stage of the cycle with cells exposed to eight sequential media alterations (i.e. PBS^1^ → AA-PBS^2^ → PBS^3^ → AA-PBS^4^ → PBS^5^ → AA-PBS^6^ → PBS^7^ → AA-PBS^8^ → AA-PBS^9^).

**Fig. 5:**
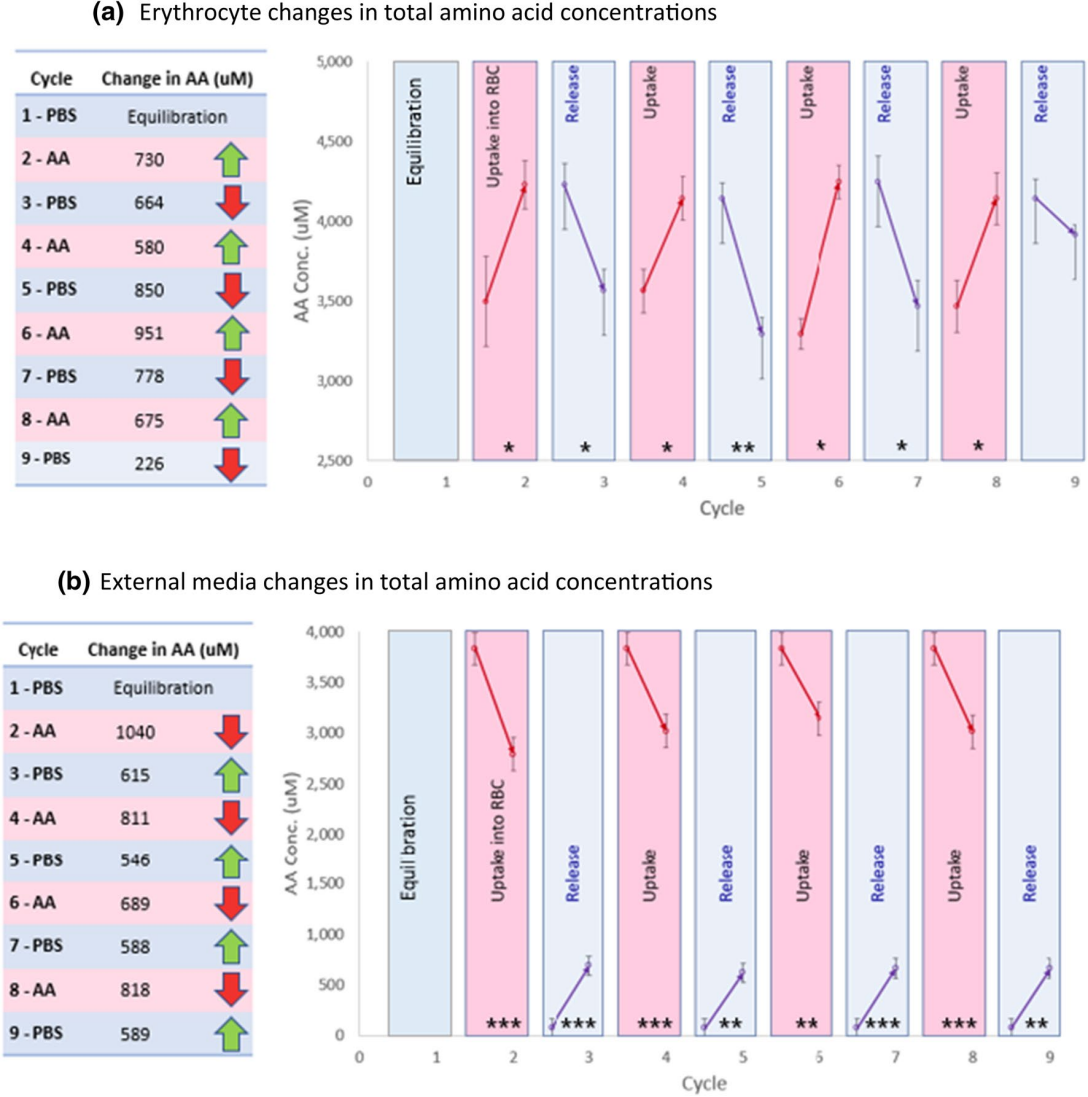
**a** The uptake and release cycles for equine erythrocytes compared with **b** the corresponding reductions in AA-PBS and increases in PBS, when the cells were exposed to nine cycles of sequential exposures to AA-PBS medium and PBS (*n* = 3). Fresh erythrocytes were subjected to a repetitive series of 2-min incubations in pre-warmed (37 °C) PBS (purple cycles 1 (equilibration), 3, 5, 7 and 9) or AA-PBS (pink cycles 2, 4, 6, and 8). Significant changes in erythrocyte concentrations of amino acids have been marked; *p < 0.05; **p < 0.01; ***p < 0.001

After the first depletion and equilibration cycle in PBS, the erythrocytes showed cyclic patterns of uptake and release when exposed to alternating solutions of high and low concentrations of amino acids, as shown in Fig. 5. When amino acids were taken up by the erythrocytes, there was a corresponding reduction in amino acids in the AA-PBS. Conversely, when amino acids were released from the erythrocytes, there was a corresponding increase in the amino acids in PBS. This pattern whereby erythrocytes gained amino acids from the AA-PBS and subsequently released them into PBS was statistically significant in all but cycle 9 (*p* < 0.05). The average increase of amino acids in erythrocytes following exposure to the AA-PBS (cycles 2, 4, 6, 8) was 734 ± 79 µM and the average corresponding reduction measured in the AA-PBS was 840 ± 73 µM. The average decrease of total amino acids in erythrocytes following release into PBS (cycles 3, 5, 7, 9) was 630 ± 140 µM and the corresponding increase measured in the PBS was 585 ± 14 µM. Together, these uptake and release quantities by the equine erythrocytes represented an average of 15% of the total amino acid load in the erythrocyte.

**Fig. 6:**
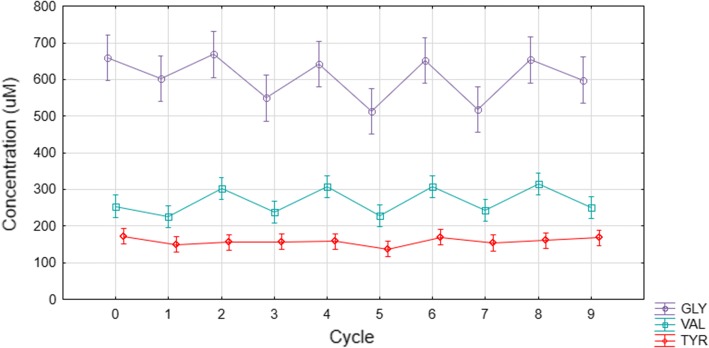
Glycine and valine uptake and release in equine erythrocytes (*n* = 3) during media cycling. Tyrosine was not included in the AA-PBS medium and the erythrocyte levels are shown as a base reference to indicate no oscillations

Analysis of individual amino acids confirmed that the amino acids that contributed significantly to the cycling trend were glycine, valine, serine, proline, phenylalanine and lysine. The alternating concentrations of glycine and valine are shown in Fig. 6 for comparison to tyrosine which had not been added to the AA-PBS and, therefore, acted as an internal reference to show no erythrocyte leakage events.

**Fig. 7:**
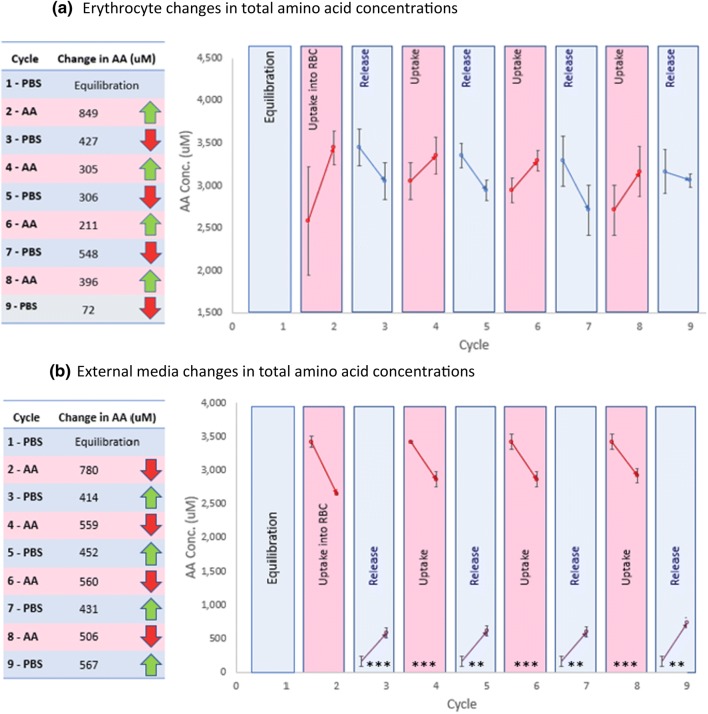
**a** The uptake and release cycles for human erythrocytes compared with **b** the corresponding reductions in AA-PBS and increases in PBS, when the cells were exposed to nine cycles of sequential exposures to AA-PBS medium and PBS (*n* = 3). Fresh erythrocytes were subjected to a repetitive series of 2-min incubations in pre-warmed (37 °C) PBS (purple cycles 1 (equilibration), 3, 5, 7 and 9) or AA-PBS (pink cycles 2, 4, 6, and 8). Significant changes in erythrocyte concentrations of amino acids have been marked; **p < 0.01; ***p < 0.001

**Fig. 8:**
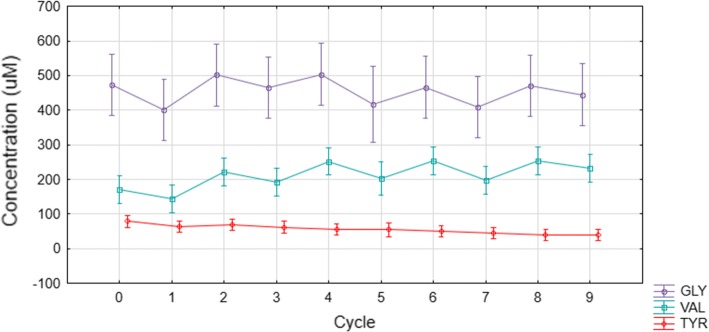
Glycine and valine uptake and release in fresh human erythrocytes (*n* = 3) during media cycling. Tyrosine was not included in the AA-PBS medium and the erythrocyte levels are shown as a base reference to indicate no oscillations

Human erythrocytes were isolated and prepared in the same way as equine erythrocytes for incubation under identical media cycling conditions. The human erythrocytes showed a similar pattern of cycling when exposed to alternating solutions of high and low concentrations of amino acids, as shown in Fig. 7. The average increase of amino acids in erythrocytes following exposure to the AA-PBS (cycles 2, 4, 6, 8) was 440 ± 141 µM and the average decrease of total amino acids in erythrocytes following release into PBS (cycles 3, 5, 7, 9) was 338 ± 102 µM. Together, these up-take and release quantities by the human erythrocytes represented an average of 16.5% of the total amino acid load in the erythrocyte. When amino acids were taken up by the erythrocytes, there was a corresponding reduction in amino acids in the AA-PBS and when amino acids were released from the erythrocytes, there was a corresponding increase in the amino acids in PBS (Fig. 7b). Certain amino acids such as valine, serine, glycine and lysine did show significant concentration alterations during each cycle (*p* < 0.01). The concentration alterations of glycine and valine have been presented alongside the concentrations of tyrosine which had not been added to the AA-PBS and, therefore, acted as an internal reference showing no oscillations with alternating exposures (Fig. 8).

## Discussion

The results clearly showed that equine and human erythrocytes were able to take up amino acids from a medium enriched with amino acids within a short time frame of less than 2 min. Furthermore, following the loading of amino acids in erythrocytes, the cells were able to release amino acids into an amino acid-free PBS medium within a 2-min period. The cells did not rapidly transfer their entire cellular content of amino acids, but instead exhibited rapid exchanges of uptake and release of smaller quantities of amino acids representing around 15% of the total amino acid load in the equine erythrocytes and 16.5% of the load in human erythrocytes.

Closer inspection of the original work by Winter and Christensen (1964) indicated that there was indeed evidence for rapid release of smaller proportions of amino acids within a 1-min time-frame. Their work was also focussed on establishing the nature of the amino acid transport systems and factors that might influence their action, rather than on determining whether the transfer rates would be appropriate for the erythrocytes to act as an inter-organ transport system. Other authors may have interpreted these findings that some amino acids had transport kinetics displaying slow and steady adjustments over prolonged periods of up to 120 min, as being too slow for the erythrocyte to act as a transport system (Brosnan 2003; Munro 1970; Scriver et al. 1971). The relevance of the rapid transfers of smaller quantities could well have been missed. This interpretation appeared to be linked to an assumption that all the amino acids in the erythrocytes would need to be unloaded in situ to serve as a delivery function. Our results were consistent with those from Winter and Christensen (1964) whereby subsequent prolonged incubation of the cells did not result in any further significant uptake or release of amino acids. Adjustments in osmotic potential would need to accompany these transfers and may involve ion transfers of Na^+^ and Cl^−^. In the current experimental context, it was not logistically possible to determine the precise time taken to complete these transfers which may have been achieved within a much shorter period of time. This result was significant in re-evaluating the potential for inter-organ transfer of amino acids.

Seven amino acid transporter systems have been identified in erythrocytes to date and are summarised in Table 3. The levels of ten out of the sixteen amino acids supplied in AA-PBS increased significantly in the equine erythrocytes after 2-min incubation in the AA-PBS and included valine, isoleucine, lysine, glycine, leucine and phenylalanine. These six amino acids are transported by either the L, y + L or y+ facilitated diffusion transport systems in the erythrocyte membrane (Deves et al. 1992; Gardner and Levy 1972; López-Burillo et al. 1985; White 1985) (Table 3). Although tyrosine can also be transported by the L system, this was not included in the medium and thus showed no change in the cytoplasmic concentrations when exposed to the AA-PBS. No specific transport systems for threonine and methionine have been identified in erythrocytes but the increase in cytoplasmic levels on incubation in the AA-PBS would be consistent with the operation of facilitated diffusion or secondary active transport systems.

**Table 3:** Amino acid transport systems identified in erythrocytes (Deves et al. 1992; Ellory et al. 1981; Ellory and Osotimehin 1984; Gardner and Levy 1972; López-Burillo et al. 1985; White 1985; Young et al. 1979)

System	Type of transport	Enhanced by	Inhibited by	Substrates
L	Facilitated diffusion	Cu+	–	Leu, Ile, Val, Phe, Tyr, Gly, Cys, Ala
y + L	Facilitated diffusion	–	–	Lys, Leu
y+	Facilitated diffusion	Na+	Sucrose	Lys, Arg, Orn
T	Facilitated diffusion	Cys	d-Trp, l-Phe, l-Try, Leu, 4-AzidoPhe, *N*-ethylmaleimide	L-Trp
ASC	Secondary active transport	Na+	Harmaline, cysteinyl-residue reagents	Ala, Ser, Cys, Gly, Gln
Gly	Secondary active transport	Na+, Cl−	Bumetanide, furosemide	Gly, Sarcosine, pro
*N*	Secondary active transport	Na+	–	Gln, Asn, His

Glutamine and histidine have been associated with the N secondary active transport system (Ellory et al. 1981; Ellory and Osotimehin 1984; Young et al. 1979). These amino acids were not taken up under our experimental conditions suggesting that the appropriate driving ion may not have been sufficiently available, or perhaps the transporters were inadvertently inhibited by a component such as cystathionine. Furthermore, energy substrates would be required to facilitate active transport processes of the ion pumps and although small endogenous supplies may have been present, none were provided in this system. No transport systems have been identified in erythrocytes for aspartic acid which showed no change in cytoplasmic concentrations following incubation in AA-PBS. Given the large differences between erythrocyte and plasma concentrations of aspartic acid reported earlier (MacLaren et al. 2000), it was proposed that a secondary active transport system would be present for this amino acid, but the system either lacked the appropriate driving ion and/or energy substrates, or the transporter could have been inhibited.

Serine has previously been associated with the ASC secondary active transport system (Ellory et al. 1981; Ellory and Osotimehin 1984; Young et al. 1979) and showed significant uptake by erythrocytes when incubated in the AA-PBS (Fig. 3, 43%). This occurred without the supply of additional energy substrates and the highly efficient uptake could suggest the presence of an additional facilitated diffusion transporter for this amino acid. Alanine was supplied in the AA-PBS at a lower concentration than the initial erythrocyte level and yet uptake was observed. This would imply the presence of a highly efficient ASC secondary active transport system which could facilitate the uptake of alanine as well as serine and glycine. The patterns of uptake of these three amino acids were very similar as shown in the Online Resource 4.

The capacity of equine and human erythrocytes to undergo repeated cycles of uptake and release, dependent on exposures to media with correspondingly higher and lower concentrations of amino acids, provided convincing evidence supporting a role for erythrocytes as an inter-organ transporter of amino acids. There are likely to be a range of factors that might regulate the rates of exchange in situ, and the provision of energy substrates on demand would potentially enable the erythrocyte to become fully functional in this capacity. This role would be consistent with the earlier observations of higher levels of amino acids in arterial erythrocytes compared with those taken from venous blood (Felig et al. 1973). The observations in the current study, where discrete packages of amino acids were released or taken up in short time frames, suggested a new hypothesis that the erythrocytes could release small bursts of amino acids as they traverse through the capillary beds during circulation. This would provide a very efficient delivery system and assist in allowing the cell to maintain osmotic homeostasis by readily adjusting nutrient and ion balance after leaving the capillary beds.

It was previously suggested that amino acids are not stored in molecular repositories in a similar way that tri-glycerides represent a store of fatty acids in adipose tissues (Brosnan 2003). Since amino acids can be retrieved through protein catabolic processes during periods of starvation, it has been assumed that this is the only endogenous source of amino acids (Brosnan 2003). However, the presence of more than half of the blood’s free amino acid in the erythrocytes, which are constantly travelling around the body, makes for an extremely efficient possible storage facility with instant access for tissues to free amino acids made available at any time. In horses, the spleen can store large quantities of healthy erythrocytes which can then be released on exercise to achieve a 50–60% increase of erythrocytes in circulation to meet additional oxygen demands (Boucher 1987). This would also translate into a 50–60% increase in the availability of free amino acids in circulation to help meet the demands of exercise metabolism. To a much lesser extent, humans can also store viable erythrocytes in the spleen with an estimated 4–5% increase in haematocrit occurring in response to exercise stimulation (Stewart and McKenzie 2002). Evidence has shown that the human spleen contains contractile proteins and could be capable of contraction to regulate its volume and release of stored erythrocytes (Tischendorf 1985). The concept of storage of viable erythrocytes in the spleen offers a further dimension to the role of erythrocytes in storing free amino acids.

These results support earlier in situ research on dogs where it was suggested that the liver could restock erythrocytes with amino acids derived from GIT-absorption and endogenous catabolism for distribution to the body (Elwyn et al. 1968). The plasma would return surplus amino acids as well as those released from endogenous catabolism and GIT-absorption to the liver. The monitoring of the horse blood as it progressed through its training regime also showed an unexpected response whereby erythrocyte amino acid concentrations increased significantly after entering the fast-work phase of training with its higher demands on gas exchange and maintenance of muscle condition. In contrast, there was little change in plasma composition of amino acids. These preliminary results suggested that the storage capacity of the erythrocyte could be increased in response to supporting a sustained high intensity exercise regime. In a similar context, it was noted in human studies that the advent of exercise led to increased levels of erythrocyte amino acids with no change in plasma levels (MacLaren et al. 2000). It was thus proposed that this concept of amino acid delivery to tissues by erythrocytes should be expanded to include an additional role whereby erythrocytes also act as a repository for amino acids for immediate access during high-intensity exercise. The capacity of the repository would be adjusted endogenously to meet the increasing demands encountered during high-intensity exercise training.

## Conclusions

The results from this study provided evidence that the equine and human erythrocytes could rapidly take up and release small quantities of amino acids, representing approximately 15–17% of the total erythrocyte content, on a cyclic basis when exposed to media with corresponding higher and lower concentrations of amino acids. Certain amino acids such as valine and serine were exchanged far more efficiently than other amino acids, indicating differential efficiencies for key substrates. The results provided strong evidence to support the role of erythrocytes as an inter-organ transporter of amino acids.

## Electronic supplementary material

Below is the link to the electronic supplementary material.

Supplementary file1 (PDF 298 kb)

Supplementary file2 (PDF 173 kb)

Supplementary file3 (PDF 89 kb)

Supplementary file4 (PDF 69 kb)
