# Supplementary material for: Evidence that human and equine erythrocytes could have significant roles in the transport and delivery of amino acids to organs and tissues
Source: Amino Acids. 2020 Apr 21;52(5):711–24. doi: 10.1007/s00726-020-02845-0 (PMC7246245; doi:10.1007/s00726-020-02845-0)
Supplement: Supplementary file 1 — Supplementary file1 (PDF 298 kb) [file 726_2020_2845_MOESM1_ESM.pdf]

Online Resource 1. Procedures undertaken to optimise extraction of amino acids from erythrocyte lysates.

Protocol	*Relative Ease* [1]	*Active Ion Exchange* [2]	*Added Preparation Time*	*Observations and comments*
Standard protocol<br>Addition of the reagent 1 from the EZ:Faast™ (Phenomenex® Inc.) derivatisation kits resulted in some precipitation of protein leading to clogging of ion exchange process for fractionation	✗	✗	-	Partial: concentrations were lost due to clogging of ion exchange step.<br>• Histidine not detected
1) Addition of 200µL 0.1 M HCl to precipitate protein followed by centrifugation.	✗	✗	10 min	High yields but reproducibility was low.
2) Non-acid precipitation of protein<br>a) acetonitrile/methanol/H_2O 2:2:1 (AMH) was very effective in precipitation but added more than an hour to processing times and cystathionine was lost.	✗	✓	>1 hr	Generated large precipitate with high yields but lost<br>• cystathionine.
b) 3% 5-Sulfosalicylic acid (SSA) was very effective in precipitation but added more than an hour to processing times and cystathionine was lost.	✗	✓	>1 hr	Low yields and lost<br>• cystathionine.
3) Allowing the erythrocyte lysate to settle for 5 min<br>This reduced clogging to 20% of the samples in a process run and histidine was not detected.	✓	✗	5 min	Extracts lost due to clogging of ion exchange step.<br>• Histidine not detected
4) Dilution of the EZ:Faast™ reagent 1 with H_2O (200 and 400µL)<br>This was performed to dilute the 20mM HCl in reagent 1 prior to addition of the lysate (following 5 minutes settling).	✓	✗	1-5 min	High yields for most AA. Clogging of ion exchange columns still occurred<br>• Histidine and cystathionine detected but erratic.
5) Spin column centrifugation<br>This process combined the 5 min settling, 400µL dilution of reagent 1 and then filtration by QIAgen spin columns by centrifuging at 15,000 xg for 15 minutes	✓	✓	25 min	High reproducible yields for all AA.

[1] *Subjective observation of the ease in which the haemolysate can be drawn through the ion exchange resin.*

[2] *The absence of protein aggregate becoming lodged in the ion exchange resin.*

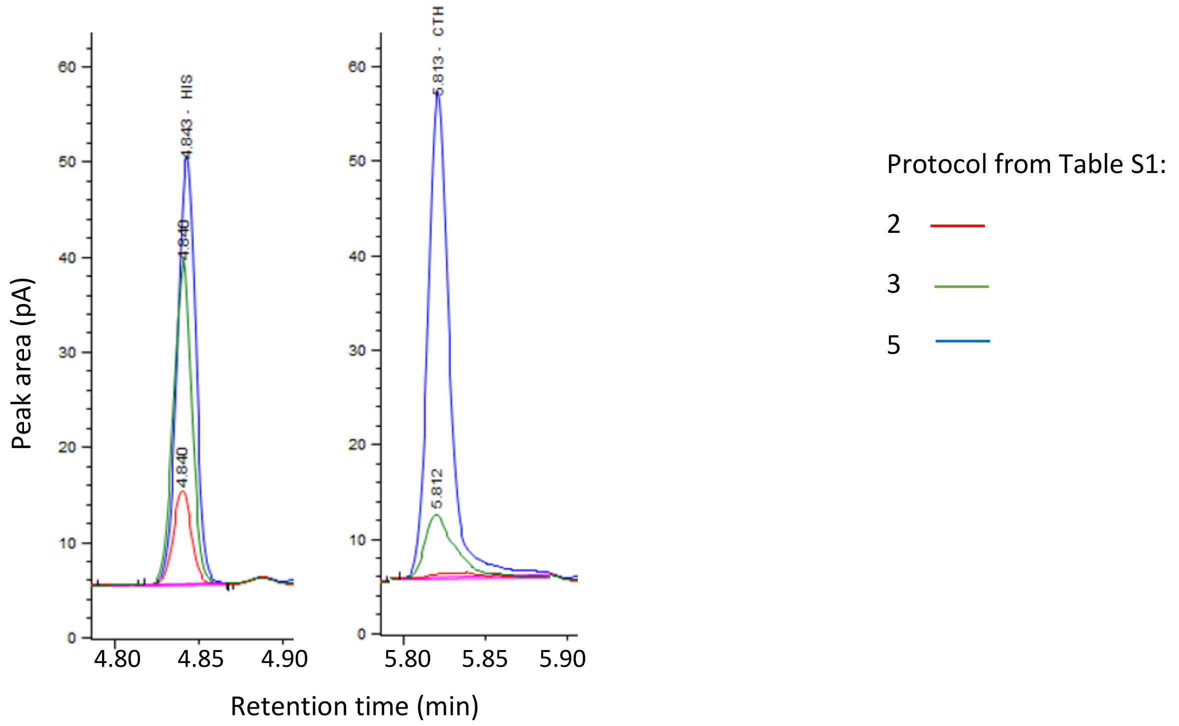

Online Resource 1 Figure 1. The chromatograms depicting the detection of histidine and cystathionine in a single sample of RBC processed with modified protocols 2 (red), 3 (green), and 5 (blue) reveals the higher yield of detection after processing with protocol 5 which is the final protocol adopted for this study.

To test the potential losses of amino acids via the additional filtration process, a standard medium of amino acids in PBS solution (AA-PBS) was prepared. Three replicates of this medium were directly analysed for amino acids and a further set was processed by filtration by the QIAgen spin columns. As shown in Figure S2, the measurements of each of the thirteen most abundant amino acids concentrations shown (as well as all other amino acids detected but not shown) confirmed that there were no significant losses of specific amino acids by introducing this extra step to the protocol. The data revealed that the assessments of individual amino acids, as well as the total cytoplasmic amino acids concentrations were not significantly different by Tukey HSD.

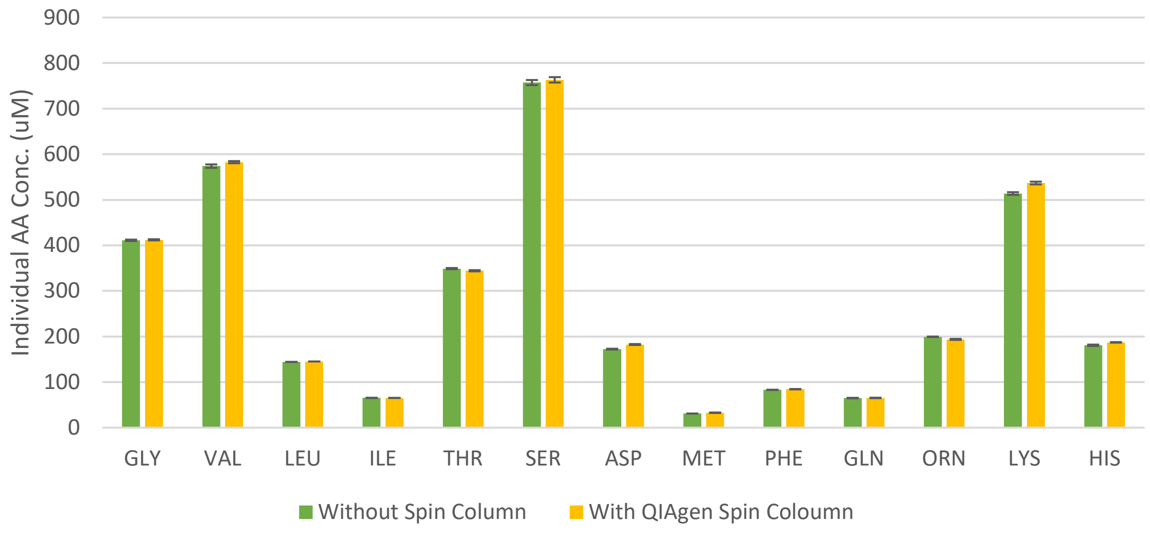

Figure S2. Analyses of samples of the AA-PBS medium were processed via the standard AA analysis protocols (green) for comparison with those processed with the additional filtration step using the QIAgen spin column (yellow).

(n = 3)
