# Supplementary figures and images for: Evidence that human and equine erythrocytes could have significant roles in the transport and delivery of amino acids to organs and tissues
Source: Amino Acids. 2020 Apr 21;52(5):711–24. doi: 10.1007/s00726-020-02845-0 (PMC7246245; doi:10.1007/s00726-020-02845-0)

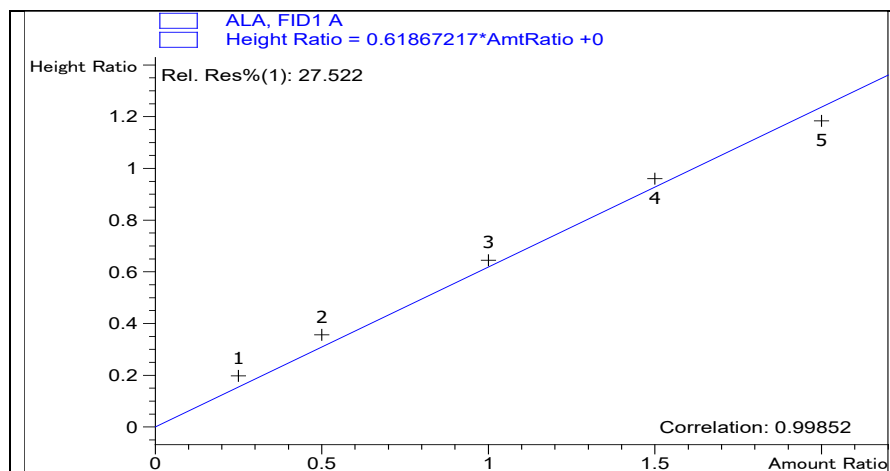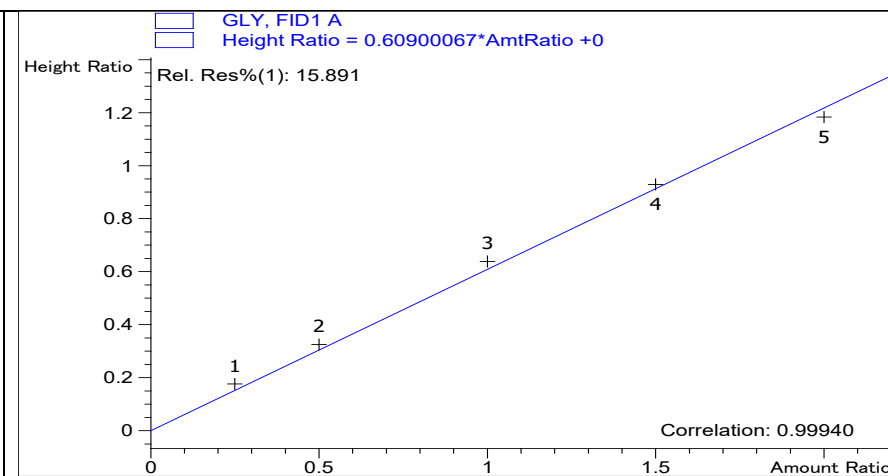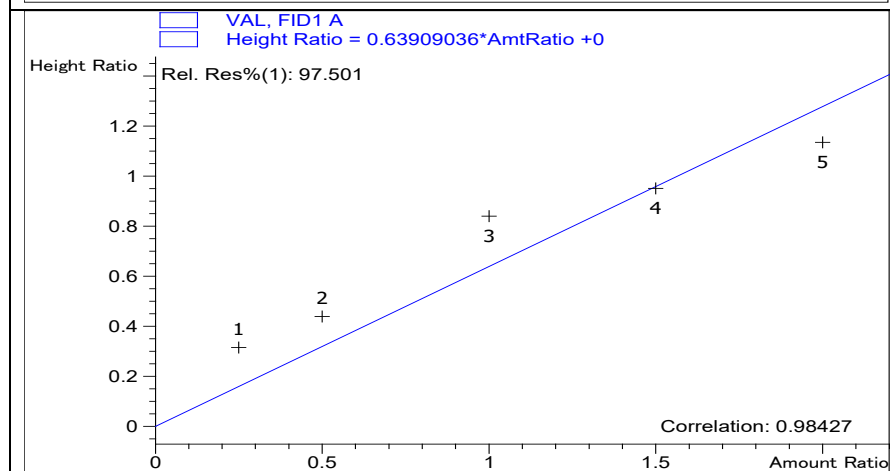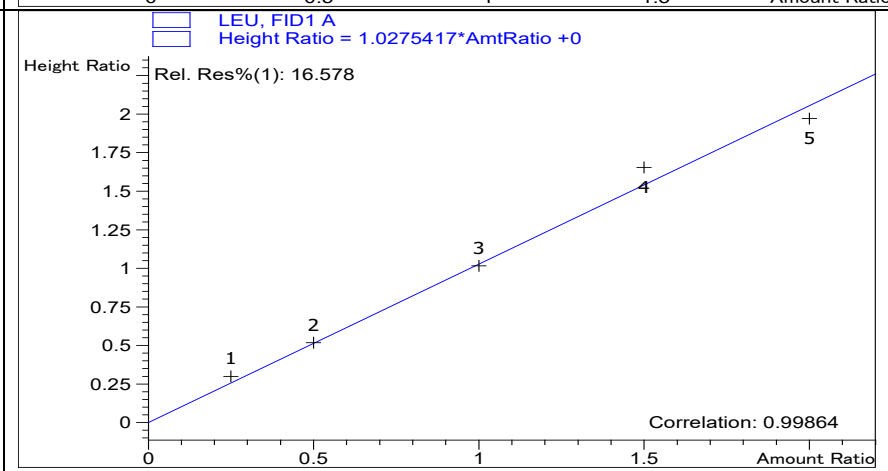

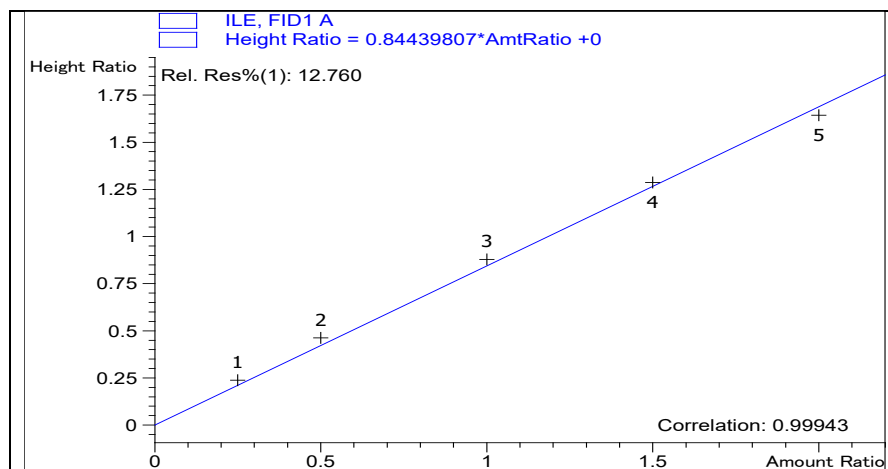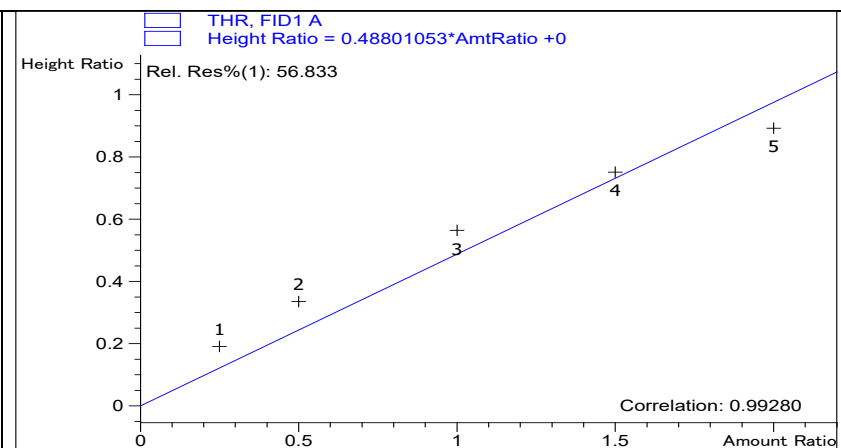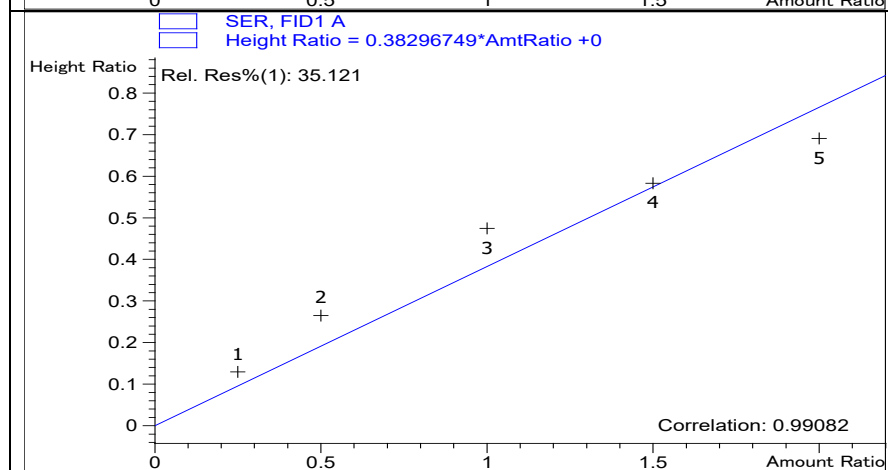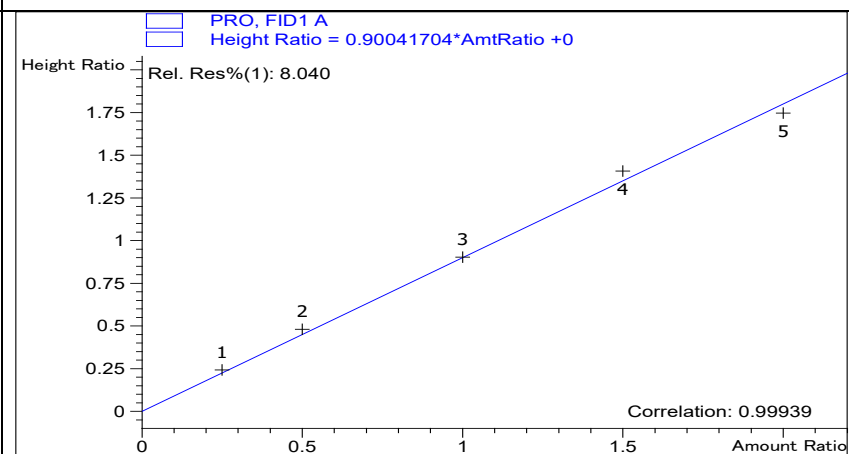

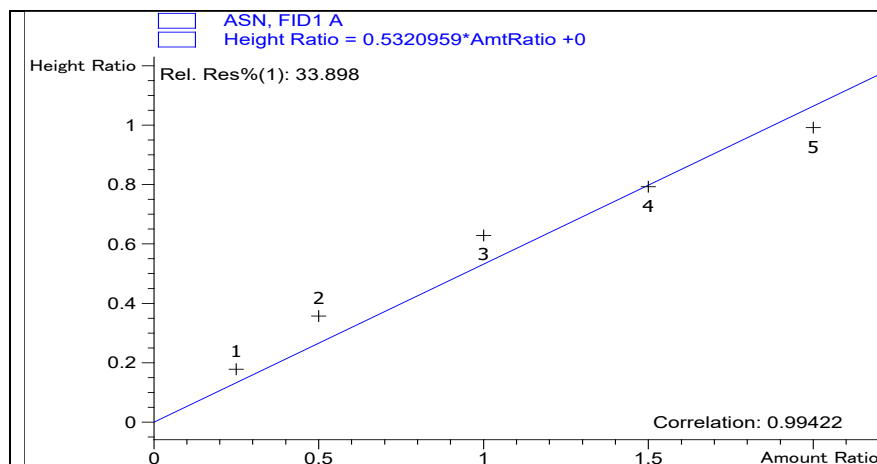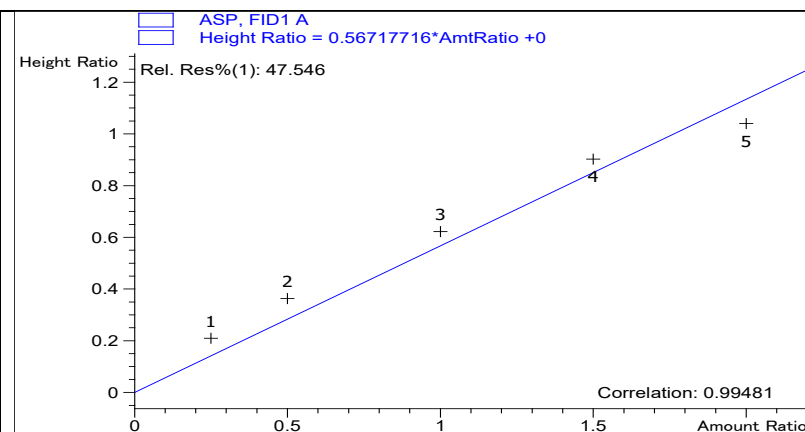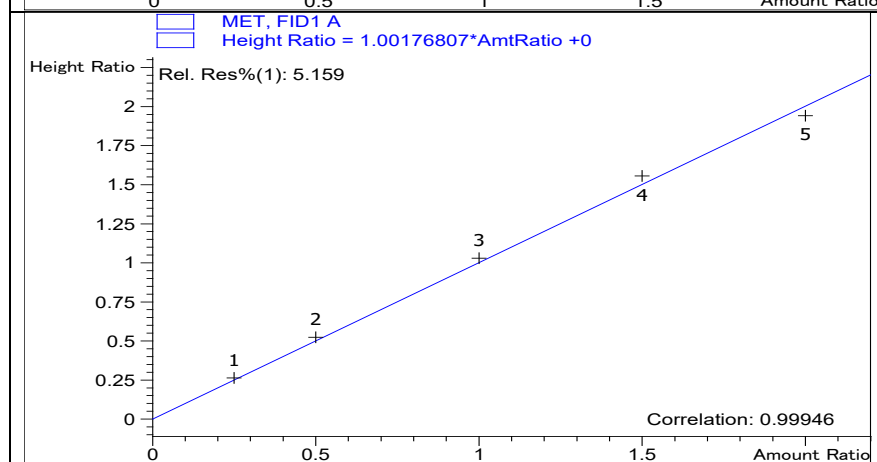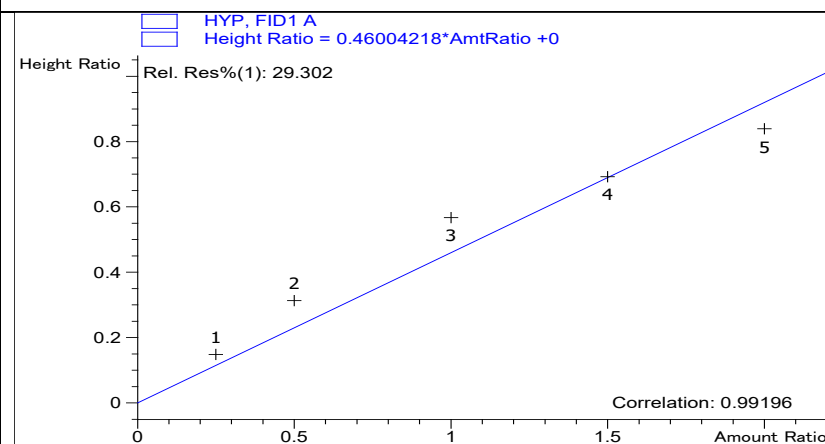

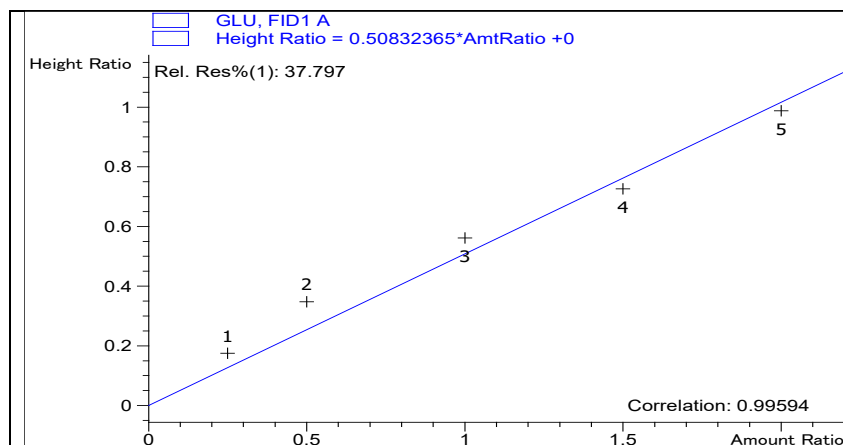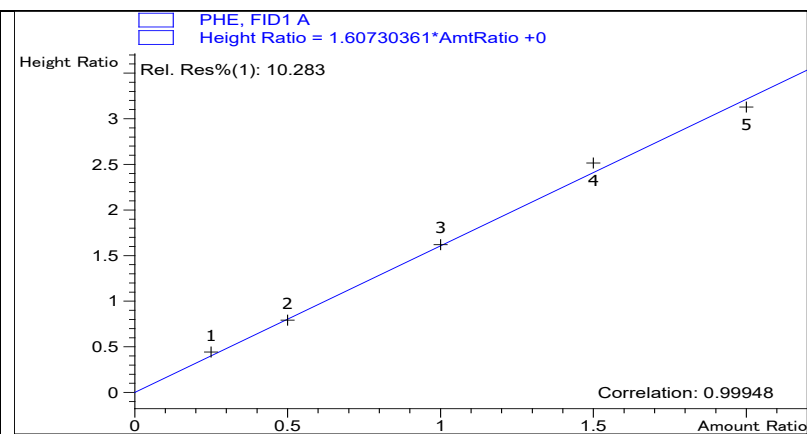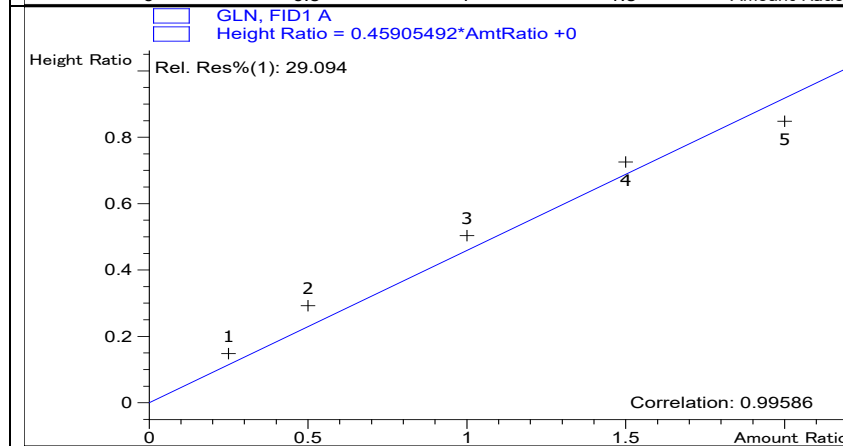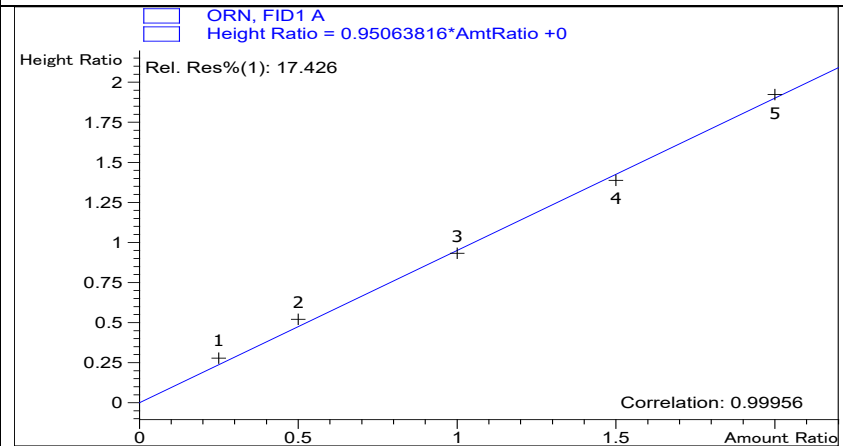

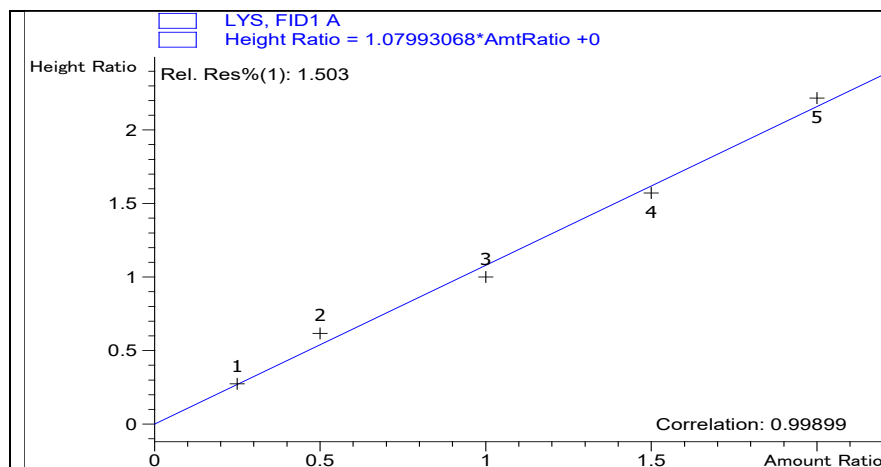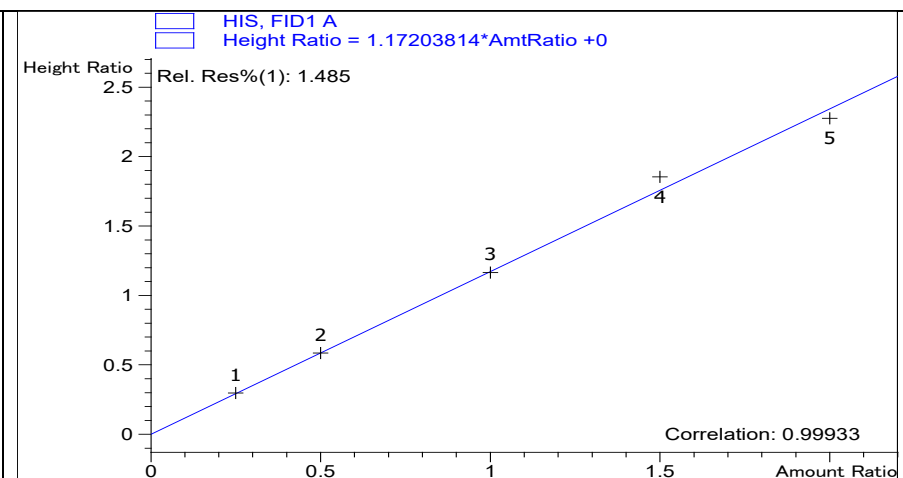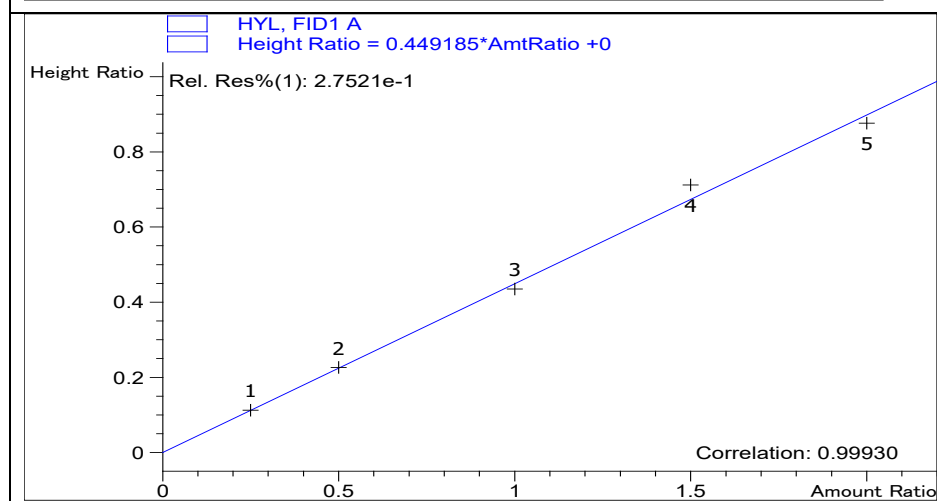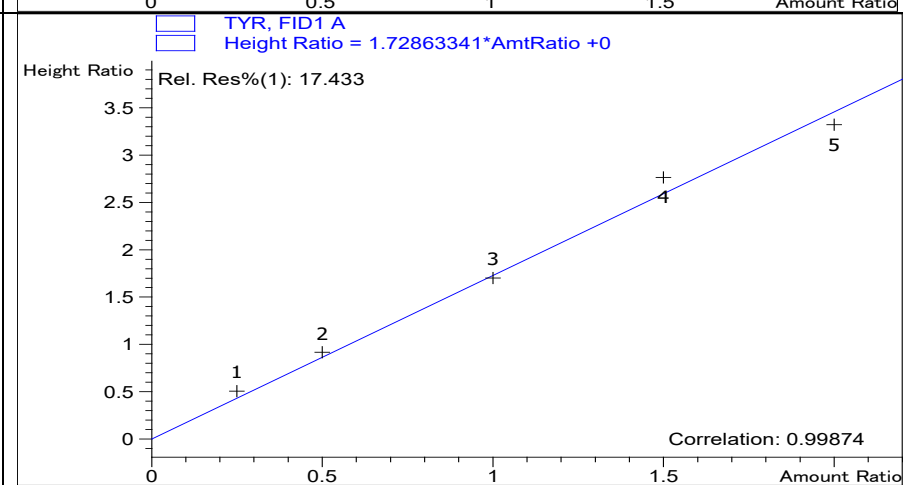

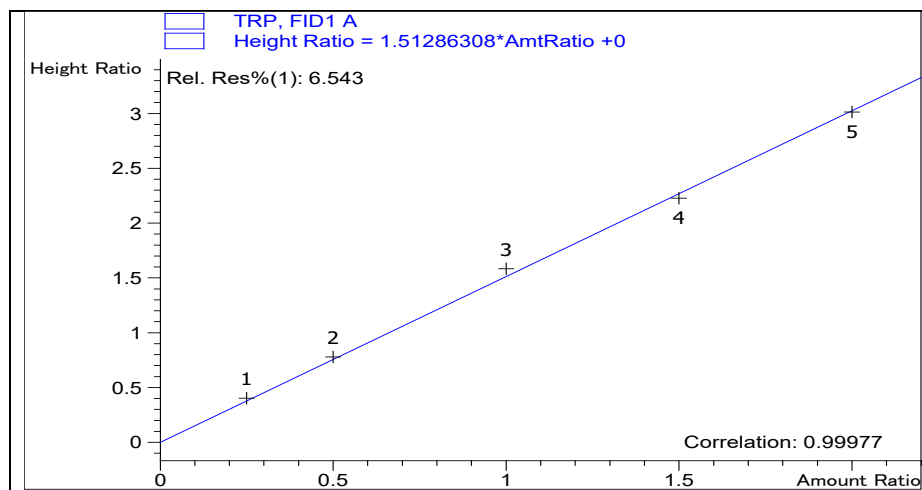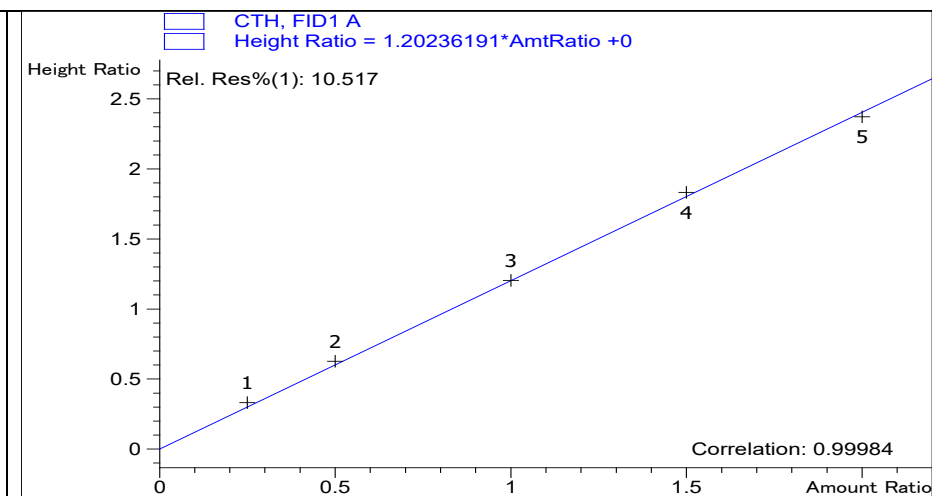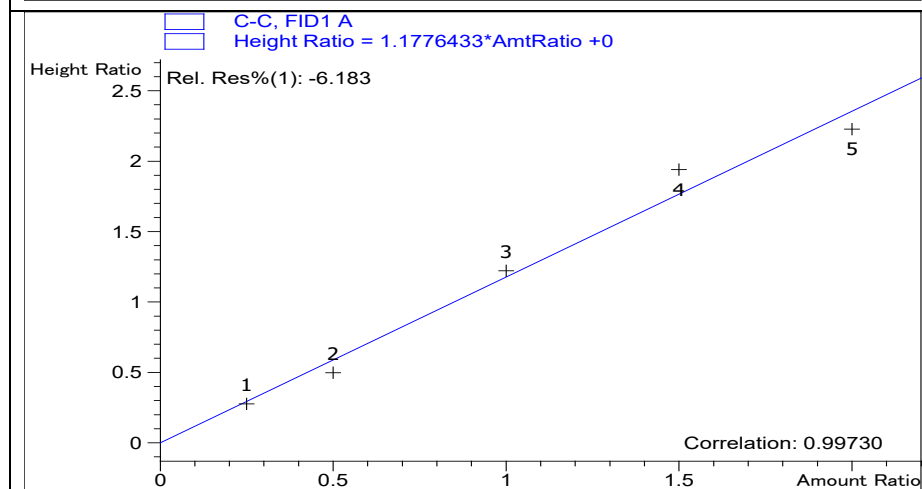

Supplement: Supplementary file 2 — Supplementary file2 (PDF 173 kb) [file 726_2020_2845_MOESM2_ESM.pdf]

Online Resource 4 Figure. Coinciding uptake and release of alanine with glycine, and serine.

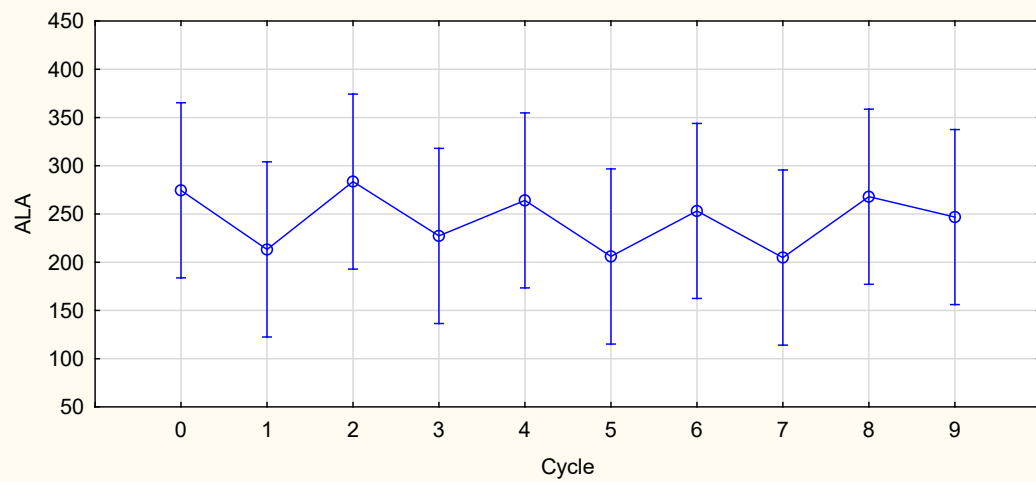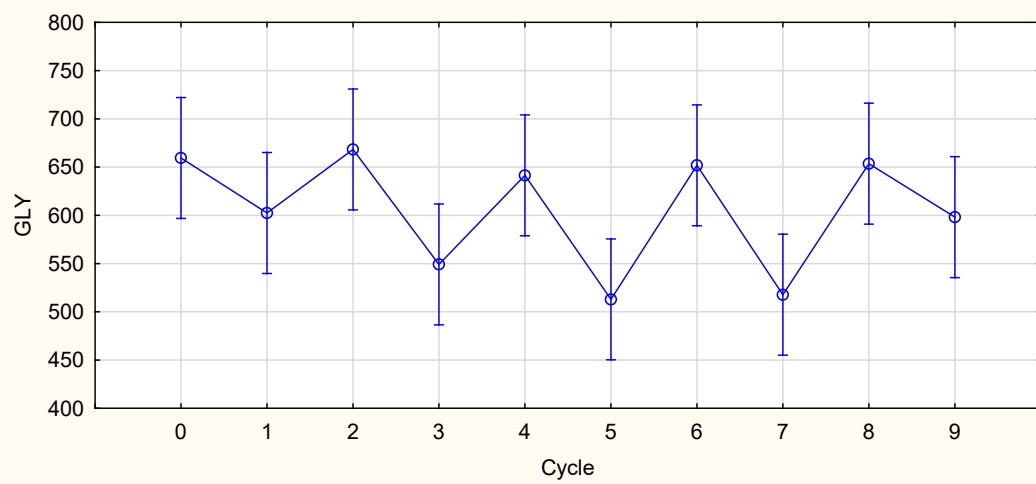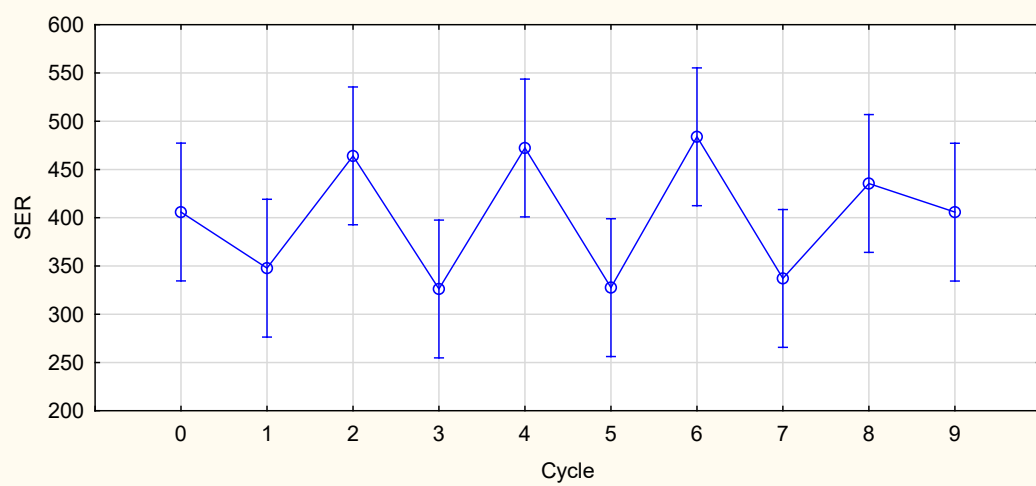

Supplement: Supplementary file 4 — Supplementary file4 (PDF 69 kb) [file 726_2020_2845_MOESM4_ESM.pdf]
