# Supplementary material for: Evidence that human and equine erythrocytes could have significant roles in the transport and delivery of amino acids to organs and tissues
Source: Amino Acids. 2020 Apr 21;52(5):711–24. doi: 10.1007/s00726-020-02845-0 (PMC7246245; doi:10.1007/s00726-020-02845-0)
Supplement: Supplementary file 3 — Supplementary file3 (PDF 89 kb) [file 726_2020_2845_MOESM3_ESM.pdf]

Online Resource 3. Comparison of amino acid levels measured in the current study by GC-FID for horses and humans, compared with those measured in previous studies with liquid chromatography techniques.

Amino Acid	Average concentration in Standardbred horse (µM) Current study	Concentration Thoroughbred horse (µM) Hanzawa and Watanabe 1991 LC Average	Average concentration in Human (µM) Current study	Concentration Humans (µM) Hagenfeldt and Arvidsson 1980 LC	Concentration Humans (µM) Agli et al 1998 Ion exchange chromatography	Concentration Humans (µM) MacLaren et al 1997 HPLC	
						Group 1	Group 2
ALA	275	450	474	256	448	1,107	1,471
ARG				19	42	40	19
ASP	123	88	79	315	2,845*	484	451
ASN	82	68	82	127			
CTH	0		0		0		
GLN	79		305	466	592		
GLU	154	63	118	238	155	412	574
GLY	587	336	473	326	527	1,222	1,589
HIS	107	299	41	81	90	342	439
ILE	59	36	27	26	89	115	140
LEU	108	115	70	43	165	498	608
LYS	303	2660*	83	124	219	489	648
MET	24		0	nd	28	128	186
ORN	165	795	109	117	262	257	342
PHE	43	44	41	20	95	209	307
PRO	318		165	123			
SER	448	413	163	145	219	466	581
THR	331	467	172	103	216	352	432
TRP	23		0		37	95	
TYR	172	292	78	46	109	153	251
VAL	439	180	170	76	221	429	582

*These values appear unusually high.

References:

Agli, A.N., A. Schaefer, B. Geny, F. Piquard, and P. Haberey. 1998. Erythrocytes participate significantly in blood transport of amino acids during the post absorptive state in normal humans. *Eur J Appl Physiol Occup Physiol*. 78:502-508.

Hagenfeldt L. and Arvidsson A. 1980. The distribution of amino acids between plasma and erythrocytes. *Clinica Chimica Acta* 100:133-141.

Hanzawa K. and Watanabe S. 1991. Free amino acid contents of horse red cells in relation to AR and BA systems: Analysis by highspeed liquid chromatography with Li-type column. *Anim. Sci. Technol.* (Jpn) 63(3):249-255.

MacLaren, D.P., A.M. Nevill, C.D. Thake, I.T. Campbell, E. Cheetham, M.A. Keegan, C. Lane, and N.B. Roberts. 2000. Human erythrocyte and plasma amino acid concentrations during exercise. *Med Sci Sports Exerc*. 32:1244-1249.
